# The ATG5-binding and coiled coil domains of ATG16L1 maintain autophagy and tissue homeostasis in mice independently of the WD domain required for LC3-associated phagocytosis

**DOI:** 10.1080/15548627.2018.1534507

**Published:** 2018-11-07

**Authors:** Shashank Rai, Maryam Arasteh, Matthew Jefferson, Timothy Pearson, Yingxue Wang, Weijiao Zhang, Bertalan Bicsak, Devina Divekar, Penny P. Powell, Ronald Naumann, Naiara Beraza, Simon R. Carding, Oliver Florey, Ulrike Mayer, Thomas Wileman

**Affiliations:** a Norwich Medical School, University of East Anglia, Norwich, Norfolk, UK; b Max-Planck-Institute of Molecular Cell Biology and Genetics, Dresden, Germany; c Quadram Institute Bioscience, Norwich, Norfolk, UK; d Signalling Programme, Babraham Institute, Cambridge, UK; e School of Biological Sciences, University of East Anglia, Norwich, Norfolk, UK

**Keywords:** ATG16L1, brain, LC3-associated phagocytosis, sequestosome 1/p62 inclusions, tissue homeostasis, WD domain, WIPI2

## Abstract

Macroautophagy/autophagy delivers damaged proteins and organelles to lysosomes for degradation, and plays important roles in maintaining tissue homeostasis by reducing tissue damage. The translocation of LC3 to the limiting membrane of the phagophore, the precursor to the autophagosome, during autophagy provides a binding site for autophagy cargoes, and facilitates fusion with lysosomes. An autophagy-related pathway called LC3-associated phagocytosis (LAP) targets LC3 to phagosome and endosome membranes during uptake of bacterial and fungal pathogens, and targets LC3 to swollen endosomes containing particulate material or apoptotic cells. We have investigated the roles played by autophagy and LAP *in vivo* by exploiting the observation that the WD domain of ATG16L1 is required for LAP, but not autophagy. Mice lacking the linker and WD domains, activate autophagy, but are deficient in LAP. The LAP^−/-^ mice survive postnatal starvation, grow at the same rate as littermate controls, and are fertile. The liver, kidney, brain and muscle of these mice maintain levels of autophagy cargoes such as LC3 and SQSTM1/p62 similar to littermate controls, and prevent accumulation of SQSTM1 inclusions and tissue damage associated with loss of autophagy. The results suggest that autophagy maintains tissue homeostasis in mice independently of LC3-associated phagocytosis. Further deletion of glutamate E230 in the coiled-coil domain required for WIPI2 binding produced mice with defective autophagy that survived neonatal starvation. Analysis of brain lysates suggested that interactions between WIPI2 and ATG16L1 were less critical for autophagy in the brain, which may allow a low level of autophagy to overcome neonatal lethality.

**Abbreviations**: CCD: coiled-coil domain; CYBB/NOX2: cytochrome b-245: beta polypeptide; GPT/ALT: glutamic pyruvic transaminase: soluble; LAP: LC3-associated phagocytosis; LC3: microtubule-associated protein 1 light chain 3; MEF: mouse embryonic fibroblast; NOD: nucleotide-binding oligomerization domain; NADPH: nicotinamide adenine dinucleotide phosphate; RUBCN/Rubicon: RUN domain and cysteine-rich domain containing Beclin 1-interacting protein; SLE: systemic lupus erythematosus; SQSTM1/p62: sequestosome 1; TLR: toll-like receptor; TMEM: transmembrane protein; TRIM: tripartite motif-containing protein; UVRAG: UV radiation resistance associated gene; WD: tryptophan-aspartic acid; WIPI: WD 40 repeat domain: phosphoinositide interacting

## Introduction

Autophagy generates autophagosomes that deliver cytoplasmic material to lysosomes for degradation. Macroautophagy, hereafter referred to as autophagy, is activated during starvation and provides a short-term supply of amino acids to sustain protein synthesis and energy production. At the same time, basal autophagy helps to prevent tissue damage during development and ageing by reducing the accumulation of damaged proteins and organelles [1,2]. Autophagosome formation involves the recruitment of LC3 from the cytosol to the limiting membrane of the phagophore where it provides a binding site for autophagy cargoes, and facilitates fusion with lysosomes. Recent studies [3] show that LC3 is also recruited to membranes by an autophagy-related pathway called LC3-associated phagocytosis (LAP). LAP is activated by TLR signalling and NADPH oxidase during phagocytosis of fungal and bacterial pathogens, and results in attachment of LC3 to the cytosolic side of the phagosome membrane where it facilitates phagosome maturation [4]. A similar LAP-like noncanonical autophagy pathway also operates in nonphagocytic cells resulting in recruitment of LC3 to single-membraned endolysosomal compartments during entosis, micropinocytosis [5] and following lysosomotropic drug treatment [6,7], and may occur during the uptake of particulate material or apoptotic cells [8–11].

Autophagy and LAP provide 2 different pathways for removing unwanted material from cells, but the relative roles played by each pathway in reducing tissue damage and maintaining homeostasis *in vivo* are not clear. Autophagy-null mice die shortly after birth because they are unable to adapt to the starvation that follows loss of placental nutrition [12]. Mice with tissue-specific loss of autophagy survive, but the tissues lacking autophagy invariably accumulate ubiquitin-positive inclusions containing protein aggregates and show signs of inflammation and tissue damage. These studies have inactivated autophagy genes such as *Atg3, Atg5, Atg7, Atg12 and Atg16L1* [12–16] that are essential for both autophagy and LAP, making it impossible to determine if tissue damage results from loss of autophagy, or LAP, or both. Studies on LAP *in vivo* have focused on inactivation of RUBCN/Rubicon in myeloid cells [3,4]. rubcn^−/-^ myeloid cells are LAP-deficient and show defects in clearance of bacterial and fungal pathogens and dying and apoptotic cells. These mice also have elevated levels of inflammatory cytokines, and eventually develop an autoimmune disease that resembles systemic lupus erythematosus (SLE) [4,17]. While suggestive of a role for LAP in preventing inflammation and autoimmunity, the targeted loss of RUBCN from myeloid cells does not inform on the role played by LAP in non-myeloid cell types *in vivo*.

**Figure 1.**
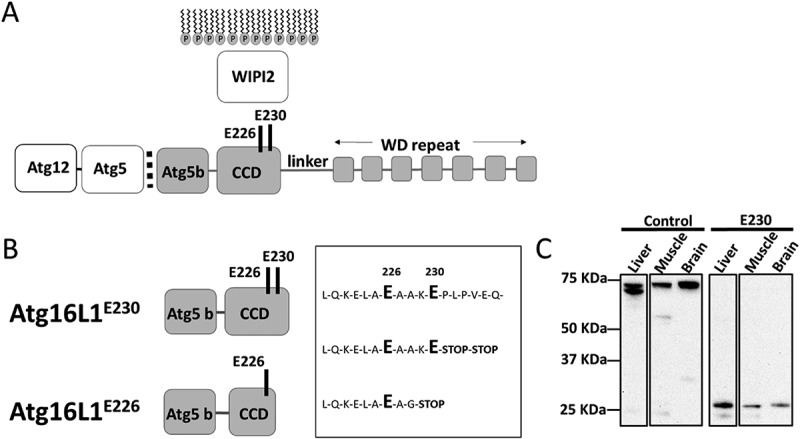
Generation of mice lacking the WD and linker domains of ATG16L1. (A) Domain structure of ATG16L1. The N-terminal ATG5 binding domain (ATG5 b) binds the ATG12–ATG5 conjugate. The coiled-coil domain (CCD) binds WIPI2 through glutamates E226 and E230. A linker domain separates the CCD from the 7 WD repeats of the WD domain. (B) Sites of stop codons. *atg16l1^E230^*; 2 stop codons were inserted into exon 6 immediately after glutamate E230 to preserve binding sites for WIPI2, but prevent translation of the linker and WD domain. *atg16l1^E226^*; an unexpected recombination inserted a glycine residue at position 228. (C) Tissue lysates were separated by SDS-PAGE and transferred to nitrocellulose membranes. Separate membranes sections were analyzed by western blot using antibody specific for ATG16L1.

The recruitment of LC3 to membranes during both autophagy and LAP requires the E3 ligase-like activity of the ATG12–ATG5-ATG16L1 complex, which covalently binds LC3 to membranes. ATG16L1 contains an N-terminal domain that binds the ATG12–ATG5 conjugate (Figure 1), followed by a coiled-coil domain (CCD) that binds WIPI2 [18]. WIPI2 brings the ATG12–ATG5-ATG16L1 complex to phagophore membranes allowing conjugation of LC3 to phosphatidylethanolamine [19]. In higher eukaryotes, a linker region attaches the CCD to a large C-terminal WD (tryptophan-aspartic acid) domain containing 7 WD repeats folded into a circular β-propeller [20,21]. Recent studies show that the WD domain of ATG16L1 is required for LAP [22]. This allowed us to generate LAP-deficient mice by inserting 2 stop codons in frame at the end of the CCD of the *Atg16L1* gene (Figure 1). This mutation, called *atg16l1^E230^*, allowed translation of the glutamate E226 and E230 residues in the CCD required for WIPI2 binding and autophagy [18], but prevented translation of the linker region and WD domain required for LAP. In a second mouse, called *atg16l1^E226^*, an unexpected recombination removed the E230 glutamate residue required for WIPI2 binding. This mouse survived post-natal starvation but was defective in autophagy and LAP in all tissues. We have used these 2 mouse strains to determine the roles played by autophagy and LAP in reducing tissue damage and maintaining tissue homeostasis *in vivo*.

### Results

Mice lacking the WD and linker domain of ATG16L1 were generated by homologous recombination in embryonic stem cells. The targeting vector was designed to insert 2 stop codons after glutamate residue E230 in the CCD, followed by the bovine growth hormone polyadenylation site and a frt-flanked neomycin cassette. Homologous recombination was verified by Southern blotting. RNA analysis of the F1 generation established from independent chimeras showed that most mice carried the correct sequence and expressed the E230 residue required for autophagy. Surprisingly, some mice had a 14 base pair (bp) deletion at the end of the CCD, which deleted glutamate E230 and its preceding amino acid, followed by a stop codon (Figure 1 A and B). These mouse strains were termed *atg16l1^E230^* and *atg16l1^E226^*.

Expression of the truncated ATG16L1^E230^ CCD was verified by comparing western blots of tissue lysates from the *atg16l1^E230^* mice with littermate controls (Figure 1C). The ATG5 binding and CCD of ATG16L1 migrated at ~ 27 kDa compared to the ~ 70 kDa for full-length ATG16L1. As described by Mizushima et al [21], lysates from liver showed the α and β isoforms of ATG16L1 while the slower migrating β isoform predominated in muscle and brain. Full-length ATG16L1 was present in lysates obtained from littermate controls, but absent from tissues of *atg16l1^E230^* mice. It was not possible to detect the truncated CCD of the *atg16l1^E226^* mice, in whole tissue lysates. This may be because the epitope is lost, or the protein is highly unstable.

**Figure 2.**
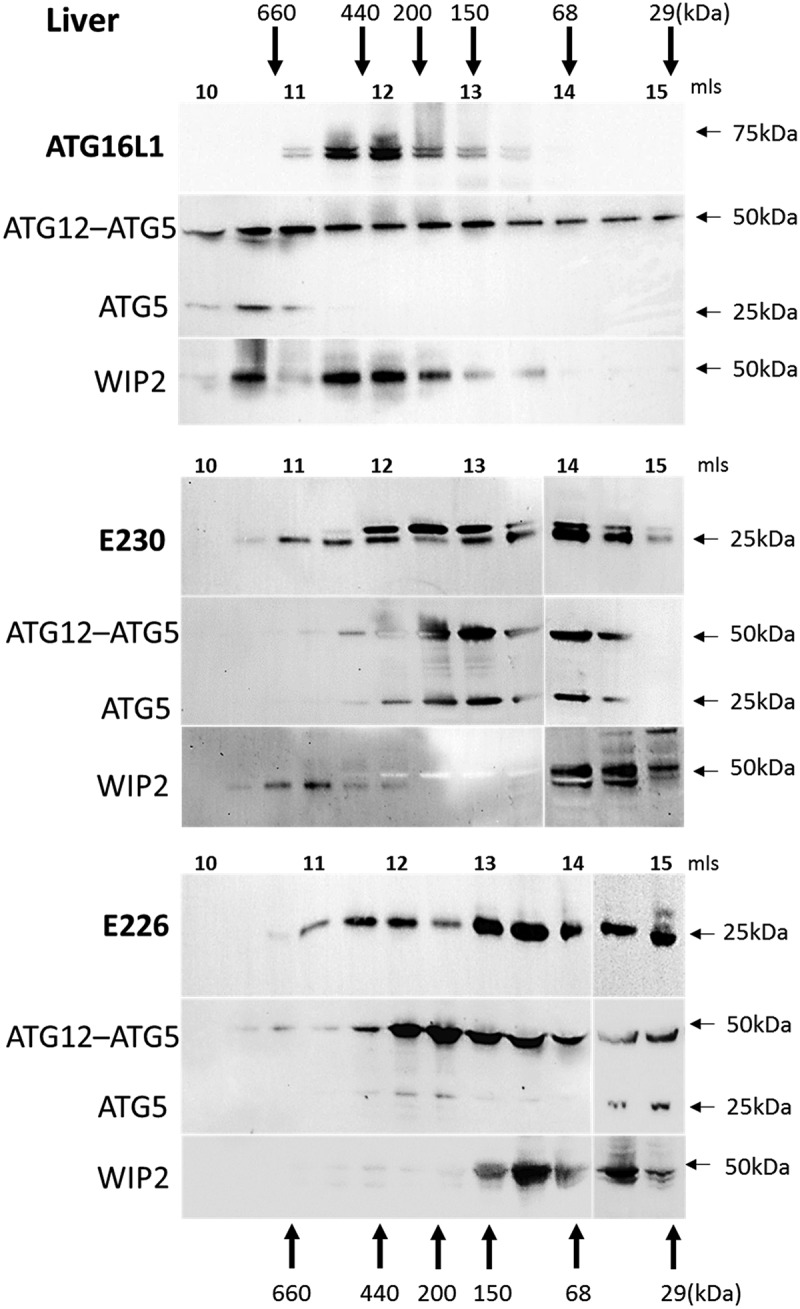
Analysis of ATG16L1 complexes in liver by gel filtration. The cytosolic fraction of liver homogenates was separated by size-exclusion chromatography on an ENrich^TM^SEC 650 column. Fraction (0.5 ml) were analyzed by immunoblot for ATG16L1, ATG5 and WIPI2 as indicated. Void volume 10 ml. Migration and elution of molecular mass standards are shown (kDa).

The size of the ATG16L1 complex generated in the mice was determined by gel filtration of cytoplasmic fractions isolated from homogenized liver (Figure 2). In control mice the α and β isoforms of ATG16L1 eluted in high molecular-mass fractions suggesting formation of a 300–600 kDa complex. Previous work has shown that elution of ATG16L1 in high molecular-weight fractions is dependent on ATG5 [21]. The presence of the ATG12–ATG5 conjugate in the same high molecular weight fractions as ATG16L1 suggested binding of ATG5 to the N-terminal ATG5-binding domain present in the CCD of ATG16L1. Full-length ATG16L1 contains the E226 and E230 glutamate residues required for WIPI2 binding, and indirect evidence for binding to WIPI2 was provided by the elution of the 49-kDa WIPI2 protein in high molecular-mass factions ranging between 150 and 600 kDa. Analysis of the *atg16l1^E230^* and *atg16l1^E226^* mice was complex because the preparation of liver lysates appeared to result in limited proteolysis of CCDs, and the possible formation of dimers and trimers resistant to dissociation during SDS polyacrylamide electrophoresis (Fig. S1). The blots in Figure 2 show the elution profile of the 27-kDa CCD and the 25-kDa proteolytic products, which co-elute with multimers at 50 and 75 kDa (Fig. S1). The bulk of the CCD in the liver of *atg16l1^E230^* mice eluted over a broad range from 50–400 kDa in fractions which also contained the ATG12–ATG5 conjugate and ATG5. As seen for control mice, WIPI2 was detected in high molecular-mass fractions eluting between 400–600 kDa; however, levels of WIPI2 were less than seen for control, and a low molecular-mass fraction was also detected between 50 and 100 kDa. The CCD of *atg16l1^E230^* mice retained binding sites for ATG5 and WIPI2, and the elution profiles were consistent with assembly of complexes containing the CCD, ATG12–ATG5 and WIPI2. Unlike whole tissue lysates, it was possible to detect the CCD of the *atg16l1^E226^* mouse in fractions eluting from the gel filtration column. The CCD eluted over a broad 30–400 kDa range in fractions that also contained ATG12–ATG5. The CCD of *atg16l1^E226^* mice lacks the E230 glutamate residue required for WIPI2. The blots show that unlike control mice and *atg16l1^E230^* mice, it was not possible to detect WIPI2 in high molecular-mass fractions, and WIPI2 eluted between 50 and 100 kDa. The results from this indirect assay based on the size of ATG16L1 complexes suggest that the CCD of *atg16l1^E226^* assembles with ATG12–ATG5 through the ATG5 binding domain, but does not bind strongly to WIPI2.

**Figure 3.**
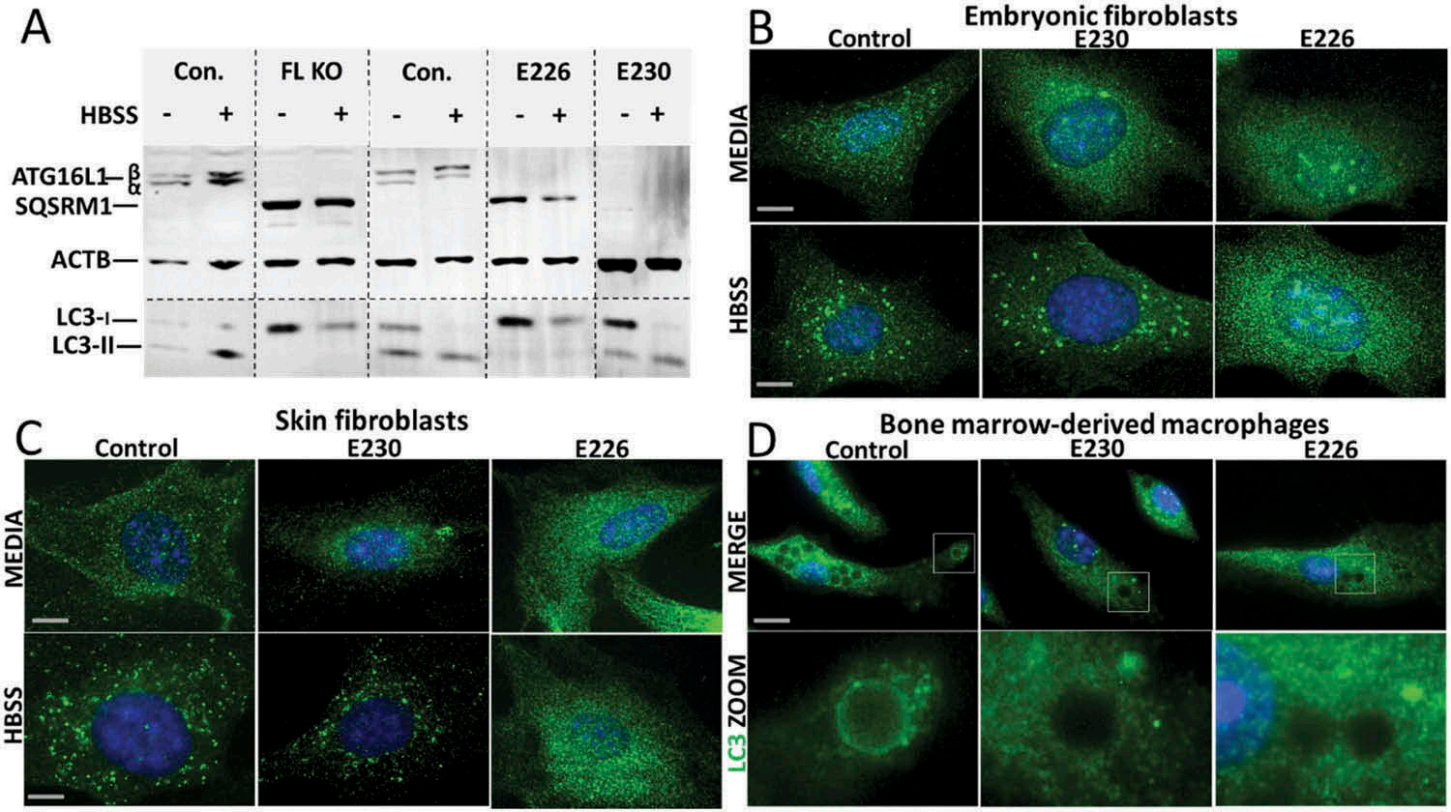
Role played by WD and linker domains of ATG16L1 during autophagy and LC3-associated phagocytosis. (A) MEFs from mice lacking ATG16L (FL KO), *atg16l1^E226^* (E226) and *atg16l1^E230^* (E230) and appropriate littermate controls were incubated in complete media or HBSS for 2 h to induce autophagy. Cell lysates were analyzed by western blot using antibodies specific for the indicated proteins. (B) MEFs or skin fibroblasts (C) from *atg16l1^E226^* (E226) and *atg16l1^E230^* (E230) mice and littermate controls were incubated in complete media or HBSS for 2 h to induce autophagy. Cells were immunostained for endogenous LC3. (D) BMDMs from *atg16l1^E226^* (E226) and *atg16l1^E230^* (E230) and appropriate littermate controls were incubated with Pam3CSK4-coupled polystyrene beads for 1.5 h in complete medium to induce LAP, and immunostained for endogenous LC3 (green). Boxed regions highlighting internalized beads are enlarged and shown in the lower panel. Magnification 63X, scale bars: 10 µm.

The ability of cells isolated from the mice to activate autophagy was tested by western blot of autophagy substrates SQSTM1/p62 (sequestosome 1) and LC3, and by following formation of LC3 puncta after starvation in Hanks balanced salt solution (HBSS) (Figure 3A-C). Mouse embryonic fibroblasts (MEFs) from mice lacking ATG16L1 (*atg16l1^−/-^*) were used as an autophagy-negative control. MEFs from control mice expressed the α and β isoforms of ATG16L1 which were absent from *atg16l1^−/-^, atg16l1^E226^ and atg16l1^E230^*MEFs, whereas *atg16l1^E230^* MEFs showed the smaller band expected at 27 kDa (data not shown). *atg16l1^−/-^* MEFs showed defects in autophagy indicated by the expression of high levels of the autophagy substrate SQSTM1, and an inability to generate lipidated LC3-II (Figure 3A) after starvation. MEFs expressing ATG16L1^E226^ also expressed high levels of SQSTM1 and were unable to generate lipidated LC3-II showing that loss of E230 resulted in defects in autophagy. This supported the observation that MEFs (Figure 3B) and skin fibroblasts (Figure 3C) from *atg16l1^E226^* mice were unable to generate LC3 puncta following starvation. In contrast, cells expressing full-length ATG16L1, or expressing the CCD but lacking the WD domain (ATG16L1^E230^) were able to activate autophagy, indicated by low basal levels of SQSTM1 and generation of LC3 puncta in response to HBSS (Figure 3B,C). Taken together, these results showed that autophagy requires the E226 and E230 glutamate residues in the CCD needed for WIPI2 binding, but, as reported previously [19], autophagy did not require the WD domain. The role played by the WD domain during LAP was analyzed by incubating bone marrow-derived macrophages (BMDMs) from the mouse strains with Pam3CSK4 (a mimic of bacterial lipopeptides)-coupled polystyrene beads to follow LC3 translocation to phagosomes (Figure 3D). LC3 was recruited to phagosomes in macrophages from control mice, but not in macrophages from autophagy-defective *atg16l1^E226^ mice, or atg16l1^E230^* mice that lack the WD domain and linker region of ATG16L1. As reported previously [22,23] these observations indicate that the WD domain is required for LAP in myeloid cells, and confirmed that the *atg16l1^E230^* mouse would provide a LAP-deficient mouse model to study the role played by LAP in maintaining tissue homeostasis *in vivo*.

### Mouse growth and survival

**Figure 4.**
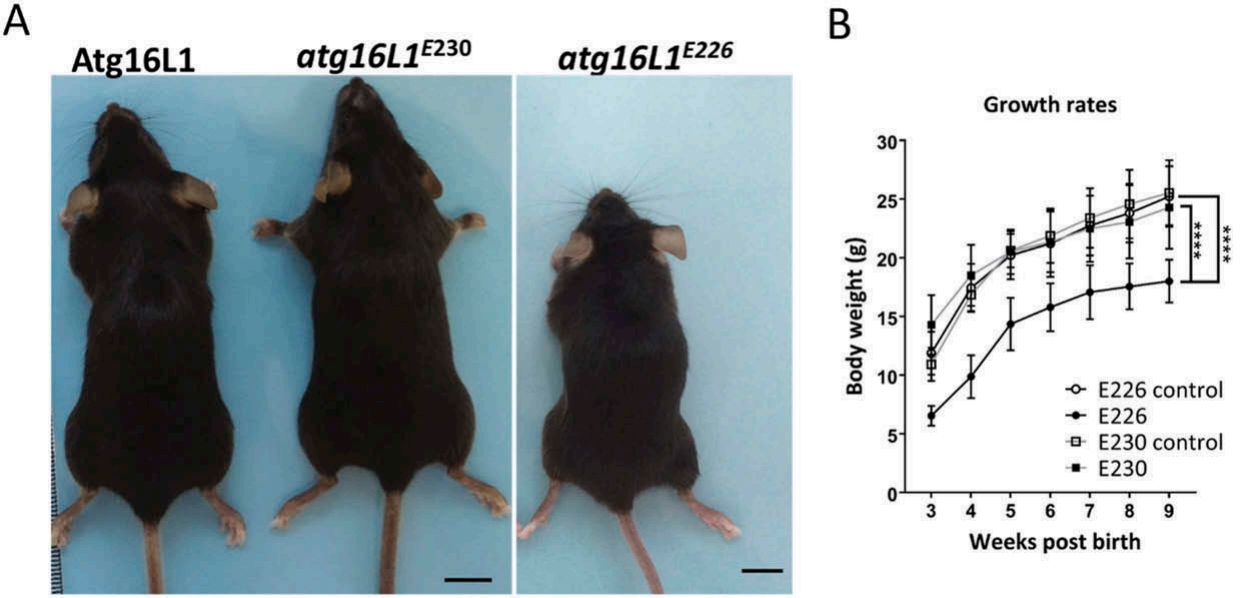
Phenotype of *atg16l1^E226^* and *atg16l1^E230^* mice. (A) Representative pictures of mice at ~2 months of age (scale bar: 1 cm). (B) Body weight of mice and littermate controls fed on chow diet. Mice were weighed at the indicted times and each point is generated from at least 6 individuals (n = 11 and 6 for E230 (*atg16l1^E230^*) and control, respectively; n = 11 and 8 for E226 (*atg16l1^E226^*) and control, respectively. Statistical analysis was done by unpaired t test. Error bars represents ±SEM. ****-P < 0.0001.

The LAP-deficient *atg16l1^E230^* mice survived the postnatal lethality seen in *atg16l1^−/-^* mice [15], and were similar in size and weight to littermate controls and grew at comparable rates (Figure 4A, B). *atg16l1^E230^* mice were born with Mendelian frequency with reproductive organs of normal size, and were fertile with a reproductive capacity comparable to controls (data not shown). The survival rate and litter sizes of *atg16l1^E230^* mice were similar to wild-type mice with life spans of at least 24 months (data not shown). The majority of *atg16l1^E226^* mice also survived postnatal lethality, but most grew slowly (Figure 4A, B) and died within 5–7 months of age.

### Inflammatory cytokines

LAP-deficient mice generated by *Lyz2/LysM-cre*-driven loss of *Rubcn* from macrophages, monocytes and neutrophils (3) develop a SLE-like syndrome characterized by an age-dependent increase in serum cytokines and eventual generation of antibodies against nuclear antigens. Serum levels of IL1B, IL12 (p70), IL13, TNF, IL6 and CCL2/MCP1 reported to be elevated in *rubcn^−/-^* mice were measured in control, *atg16l1^E230^* and *atg16l1^E226^* mice. Serum levels of IL1B, IL12 (p70), IL13, and TNF/TNF-α in *atg16l1^E230^* and *atg16l1^E230^* mice were the same as littermate controls at 8–12 and 20–24 wk. Elevated levels of IL6 and CCL2/MCP1 were detected in the autophagy-defective *atg16l1^E226^* mice, but these were not seen in control or *atg16l1^E230^* mice (Fig. S2). It was not possible to detect antinuclear antibodies able to stain the nuclei of cells in culture. The LAP^−/-^ *atg16l1^E230^* mice do not therefore appear to develop the pro-inflammatory phenotype seen for *rubcn^−/-^* mice lacking LAP in phagocytic cells.

### Liver

**Figure 5.**
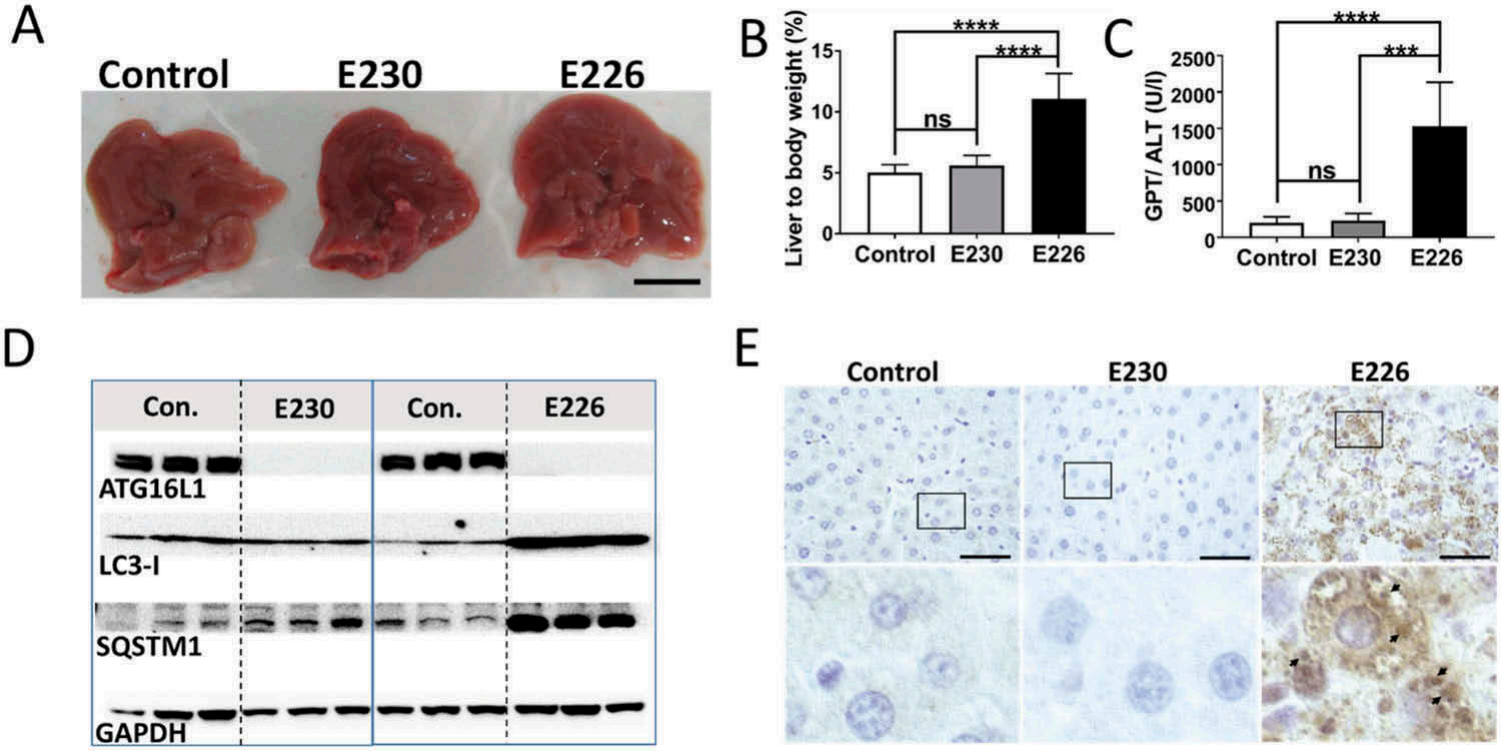
Analysis of autophagy substrates in liver. Panel (A) Representative livers at ~2 months (scale bar: 1 cm). (B) Liver weight expressed as a percentage of body weight at 2–3 months of age. E230 (*atg16l1^E230^*) n = 9, control n = 8; E226 (*atg16l1^E226^*) n = 9, control n = 7. (C) GPT/ALT in serum from mice aged between 2–3 months. E230 (*atg16l1^E230^*) n = 7, control n = 5; E226 (*atg16l1^E226^*) n = 5, v control n = 5. (D) Western blot of liver lysates from 3 representative mice. Membranes strips taken from the appropriate molecular weight range were analyzed separately using the indicted antibodies. (E) Representative histochemical sections of livers immunostained for SQSTM1. Enlarged regions of interest are shown in the lower panel. Arrows: SQSTM1 inclusions. In all figures data from littermate controls for E230 and E226 were pooled. Statistical analysis was done by unpaired t test. Error bars represents ±SEM. ****-P < 0.0001, ***-P < 0.001; ns, non-significant. Image magnification 40X, scale bars: 50 µm.

**Figure 6.**
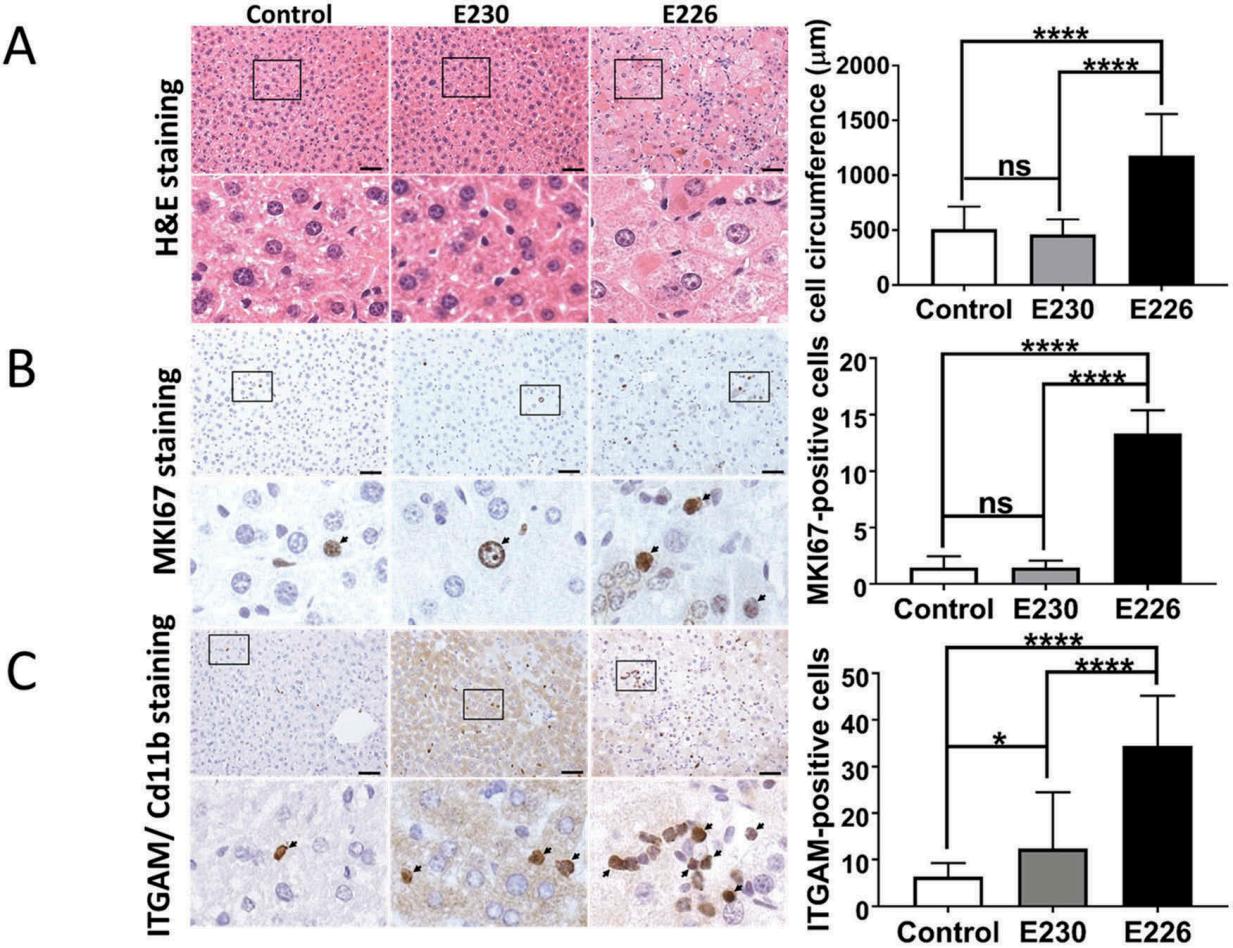
Analysis of liver homeostasis. (A) Representative images of H&E-stained sections of livers. Boxed regions of interest are enlarged in lower panels. The bar graph represents comparative circumferences of hepatocytes (n = 10) across the indicated strains (n = 3 for all the strains). (B and C) Representative histochemical sections of liver immunostained with antibodies against MKI67/Ki67 (B) or ITGAM/Cd11b (C). Regions of interest are enlarged and shown in lower panels. Arrows indicate positive staining. Bar graphs show number of positive cells (C) or percent positive cells (B). Five different zones for each liver section were analyzed (n = 3 for all the strains). Data across littermate control mice for E230 and E226 were pooled. Statistical analysis was done by unpaired t test. Error bars represents ±SEM. ****-P < 0.0001, *-P < 0.1. Magnification 20X, scale bars: 50 µm.

A preliminary survey of the internal organs of the mouse strains showed that defects were most obvious in the liver of the autophagy-defective *atg16l1^E226^* mice. The livers (Figure 5A) showed pronounced hepatomegaly with average liver weights of 2.6 g, compared to 1.6 g of control mice, and when liver weight was expressed as percentage of body weight (Figure 5B) to compensate for the smaller size of the *atg16l1^E226^* mouse, livers were more than twice the size (11%) of littermate controls (5%). These results are in line with studies of mice with targeted loss of *Atg*7 in liver [13], or the hepatomegaly observed in *atg5^−/-^* mice where *Atg5* is restored in neuronal tissue [24]. Liver damage was evident from elevated levels of serum GPT/ALT (glutamic pyruvic transaminase, soluble), a liver function marker enzyme (Figure 5C). In contrast, there was little evidence for hepatomegaly in *atg16l1^E230^* LAP-deficient mice with mean weights of 1.8 g being the comparable to littermate controls, and these mice did not show the raised serum GPT/ALT levels seen in autophagy-defective mice. Autophagy function in liver tissue was assessed by western blot of SQSTM1 and LC3, which are substrates for autophagy and are degraded in lysosomes. The signals for full-length ATG16L1 were present in blots of 3 representative livers taken from control mice. Two bands were seen, consistent with the expression of α and β isoforms of ATG16L1 in liver [21], but absent from blots of *atg16l1^E226^* and *atg16l1^E230^ mice* (Figure 5D). Liver lysates from the *atg16l1^E226^* mouse showed raised levels of LC3-I and SQSTM1 compared to control mice, suggesting impaired clearance of autophagy substrates ‘in vivo’ (Figure 5D). Immunohistological analysis showed SQSTM1 inclusions in hepatocytes, indicative of accumulation of damaged proteins and organelles in *atg16l1^E226^* mice (Figure 5E). In contrast livers from the LAP-deficient *atg16l1^E230^* mice were similar in size to littermate controls (Figure 5A), and lysates did not reveal raised levels of LC3-I and SQSTM1 (Figure 5D). Moreover, SQSTM1 inclusions were absent from hepatocytes (Figure 5E), suggesting that autophagy is active in the liver. Further analysis confirmed that hepatomegaly in *atg16l1^E226^* mice correlated with hepatocellular hypertrophy as evidenced by the enlarged circumference of hepatocytes (Figure 6A) and increased hepatocyte proliferation indicated by a 4-fold increase in MKI67/Ki67 immunostaining (Figure 6B). Liver inflammation was also evident from increased infiltration of ITGAM/CD11b-positive leukocytes (Figure 6C). In contrast, livers from the *atg16l1^E230^* mice showed little sign of damage. A comparison of the mice suggested that LAP was not required to suppress tissue damage because the liver parenchyma damage, hepatocellular hypertrophy (Figure 6A) and raised serum GPT/ALT levels (Figure 5C) seen in the *atg16l1^E226^* mice, were absent from *atg16l1^E230^* mice. Furthermore, hepatocyte proliferation (Figure 6B) was comparable to that found in littermate controls. There was evidence of low level inflammatory cell infiltration in the livers from *atg16l1^E230^* mice (Figure 6C), possibly indicating a role for LAP in reducing inflammation, but levels of ITGAM/Cd11b-positive cells were much lower than seen in livers of autophagy-defective *atg16l1^E226^* mice.

### Muscle and kidney

**Figure 7.**
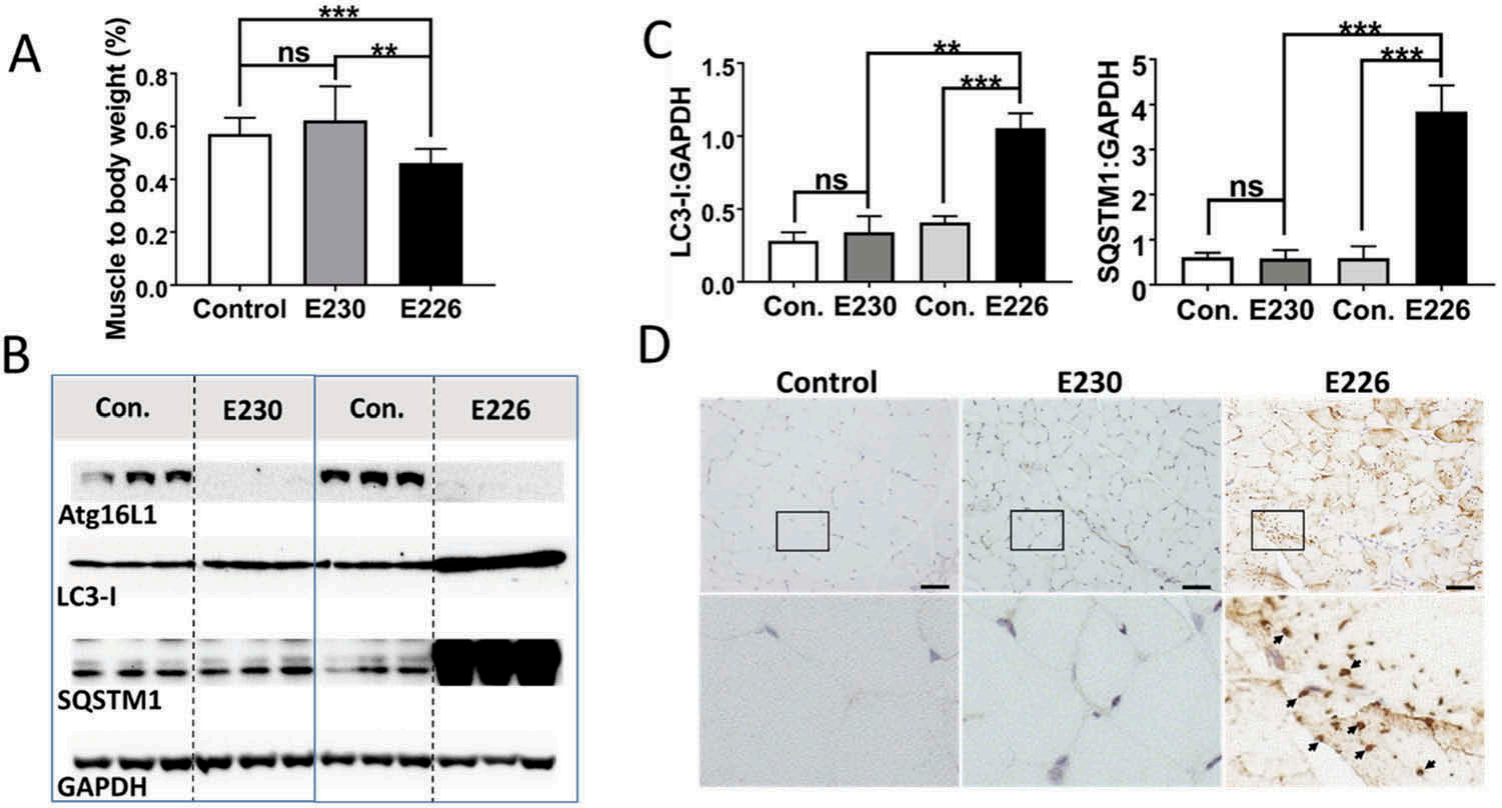
Analysis of muscle. (A) Gastrocnemius muscle weight expressed as a percentage of body weights at 2–3 months of age. E230 (*atg16l1^E230^*) n = 6, control n = 5; E226 (*atg16l1^E226^*) n = 8, control n = 6. (B) Western blot of muscle lysates from 3 representative mice. Membrane strips taken from the appropriate molecular weight range were analyzed separately by western blot using the indicted antibodies. (C) Bar graphs show levels of LC3 and SQSTM1 relative to GAPDH. (D) Histochemical sections of muscle were immunostained with antibodies against SQSTM1. Enlarged regions of interest are shown in the lower panels. Arrows: SQSTM1 inclusions. Statistical analysis was done by unpaired t test. Error bars represent ±SEM. ***-P < 0.001, **-P < 0.01; ns, non-significant. Magnification 20X, scale bar: 50 µm.

The *atg16l1^E226^* mice showed evidence of muscle wasting because the gastrocnemius muscle was significantly smaller in *atg16l1^E226^* mice when muscle weight was expressed as a percentage of body weight (Figure 7A). Lysates from muscle had high levels of SQSTM1 and LC3 (Figure 7B), and when normalized for GAPDH expression, muscle lysates from the *atg16l1^E226^* mice showed 2–3 fold increases in LC3-I, and nearly 6-fold increases in SQSTM1 compared to control mice (Figure 7C), suggesting impaired clearance of autophagy substrates ‘in vivo’. This was consistent with histological sections of muscle showing multiple SQSTM1 inclusions (Figure 7D). Morphological analysis of muscle did not, however, reveal the degenerative changes observed in mice lacking ATG7 in muscle tissue [25], such as vacuolated and centrally nucleated myofibers.

**Figure 8.**
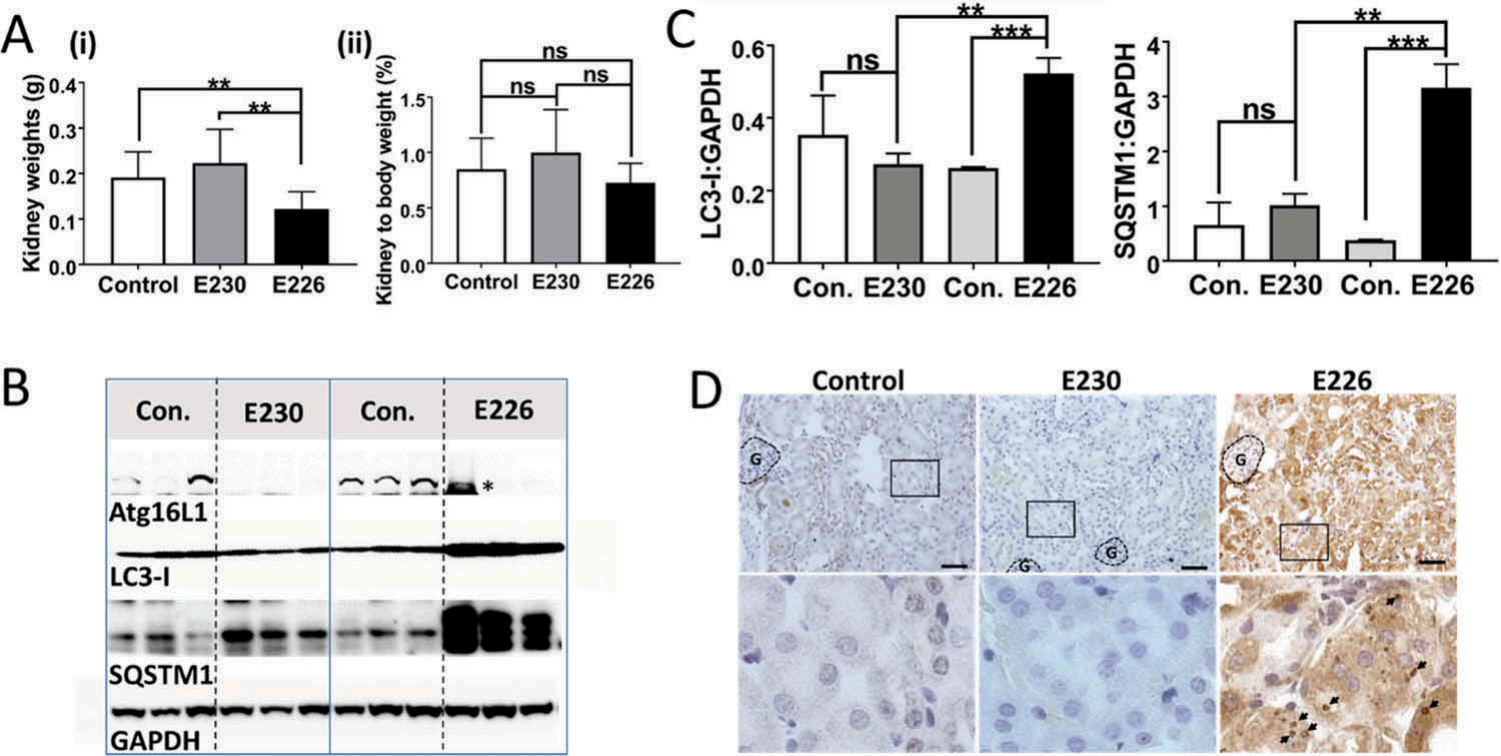
Analysis of kidney. (A) Kidney weights at 2–3 months expressed (i) directly: E230 (*atg16l1^E230^* n = 8, control n = 8, E226 (*atg16l1^E226^*) n = 7, control = 8; or as (ii) percentage body weight: E230 (*atg16l1^E230^* n = 9, control n = 8, E226 (*atg16l1^E226^*) n = 7, control = 6. (B) Western blot of kidney lysates from 3 representative mice. Membranes strips taken from the appropriate molecular weight range were analyzed separately using the indicted antibodies. (C) Bar graphs show levels of LC3 and SQSTM1 relative to GAPDH. (D) Histochemical sections of kidneys immunostained for SQSTM1. (G) Indicates glomerulus. Enlarged regions of interest are shown in lower panels. Arrows: SQSTM1 inclusions. Data from control mice were pooled. Statistical analysis was done by unpaired t test. Error bars represent ±SEM. ***-P < 0.001, **-P < 0.01; ns, non-significant. Magnification 20X, scale bars: 50 µm.

The kidneys of *atg16l1^E226^* mice weighed less than controls, but this difference was not statistically significant when expressed as a percentage of body weight (Figure 8A). Kidney lysates of *atg16l1^E226^* mice contained raised levels of LC3-I and SQSTM1 (Figure 8B and C) and histological sections of kidney showed multiple SQSTM1 inclusions (Figure 8D). We were not, however, able to find evidence of obvious kidney damage and glomerular architecture remained intact. In contrast to the autophagy-negative *atg16l1^E226^* mice, the muscle and kidney of LAP^−^deficient *atg16l1^E230^* mice were of comparable size to littermate controls (Figures 7A and 8A) and blots of tissue lysates did not show raised levels of LC3-I or SQSTM1 (Figures 7B and 8B), consistent with the absence of SQSTM1 inclusions in tissue sections (Figures 7D and 8D). As seen for liver, tissue homeostasis in kidney and muscle required autophagy, but did not require the WD domain of ATG16L1 and was, therefore maintained independently of LAP.

### Brain

**Figure 9.**
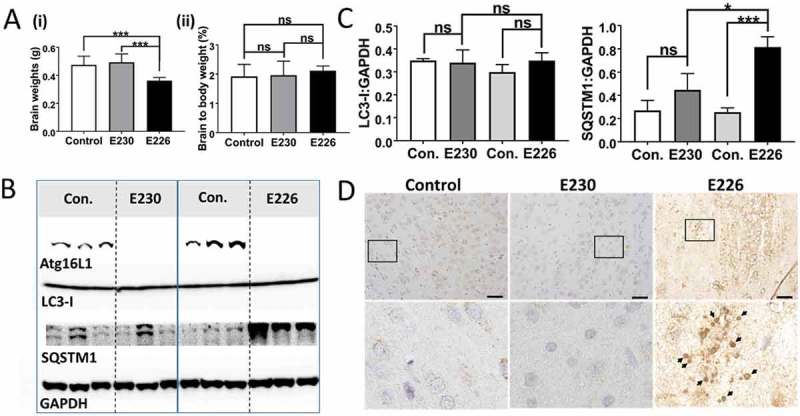
Analysis of brain. (A) Brain weights at 2–3 months expressed (i) directly: E230 (*atg161^E230^* n = 8, control n = 7, E226 (*atg16l1^E226^*) n = 7, control = 7; or (ii) as percentage body weight: E230 (*atg161^E230^* n = 8, control n = 9, E226 (*atg16l1^E226^*) n = 6, control = 5. (B) Western blot of brain lysates from 3 representative mice. Membrane strips taken from the appropriate molecular weight range were analyzed separately by western blot using the indicted antibodies. (C) Bar graphs show levels of LC3 and SQSTM1 relative to GAPDH. (D) Histochemical sections of brains were immunostained for SQSTM1. Enlarged regions of interest are shown in lower panels. Arrows indicate SQSTM1 inclusions. Data from control mice were pooled. Statistical analysis was done by unpaired t test. Error bars represents ±SEM. ***-P < 0.001, *-P < 0.1; ns, non-significant. Magnification 20X, scale bars: 50 µm.

The autophagy-defective *atg16l1^E226^* mice showed a range of neurological defects including loss of motor coordination, abnormal limb clasping and an unusual splayed gait suggesting defects in both the peripheral and central nervous system (data not shown). The neurological phenotype of autophagy-defective *atg16l1^E226^* mice mirrored the loss of motor coordination seen in mice deficient in ATG7 in the central nervous system [26]. The brains of the *atg16l1^E226^* mice were smaller than littermate controls, but there was no significant difference when brain weights were expressed as a percentage of body weight (Figure 9A). There were high levels of SQSTM1 in brain lysates (Figure 9B and C), and brain sections showed evidence of SQSTM1 inclusions (Figure 9D). In contrast, brain lysates from the LAP-deficient *atg16l1^E230^* mice had levels of LC3-I and SQSTM1 similar to controls (Figure 9B and C), and brains lacked SQSTM1 inclusions (Figure 9D). Although motor coordination appeared normal in the majority of the *atg16l1^E23^*^0^ mice, some developed head tilt suggesting defects in the inner ear or possible ear infection.

**Figure 10.**
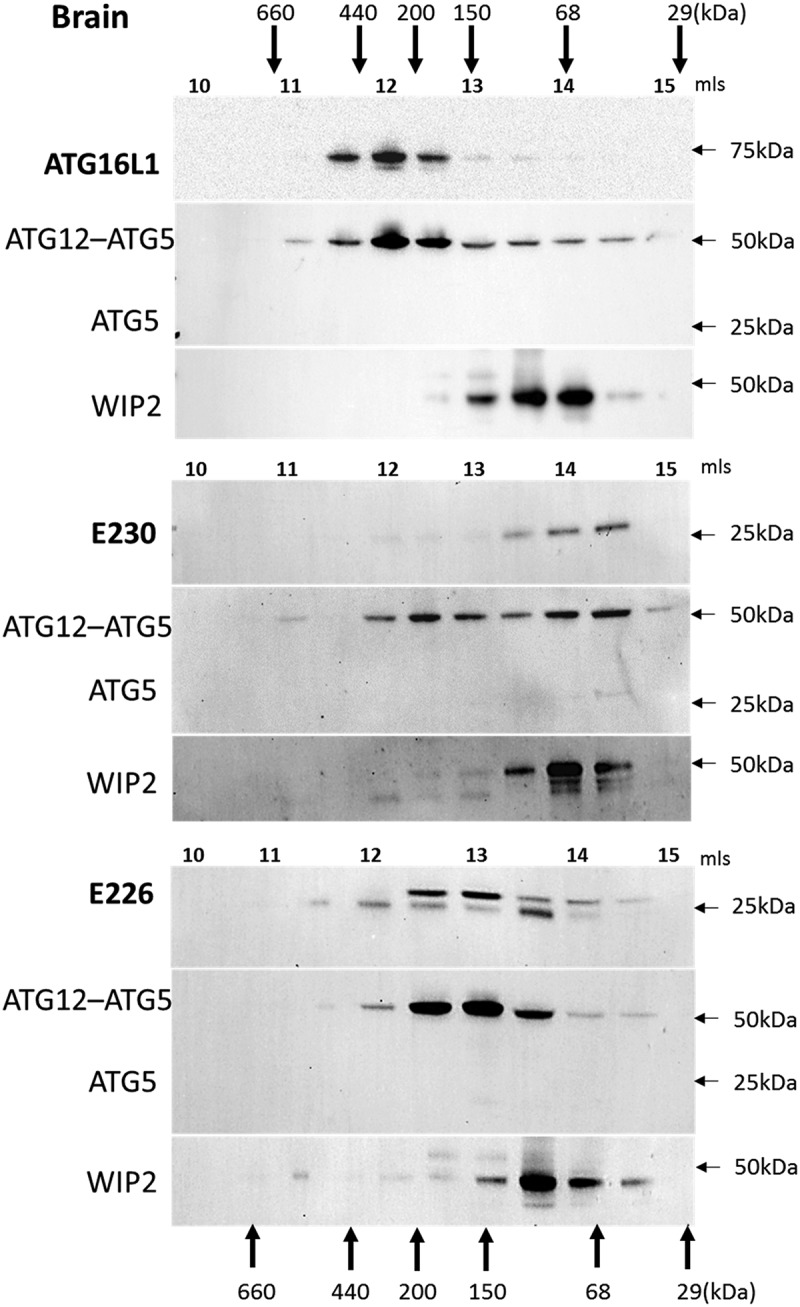
Analysis of ATG16L1 complexes in brain by gel filtration. The cytosolic fraction of brain homogenates was separated by size-exclusion chromatography on an ENrich^TM^SEC 650 column. Fraction (0.5 ml) were analyzed by immunoblot for ATG16L1, ATG5 and WIPI2 as indicated. Void volume 10 ml. Migration and elution of molecular mass standards are shown (kDa).

Mice with systemic loss of ATG16L1 from all tissues (*atg16l1*^−/-^) die at birth from a suckling defect [15]. This raised the question of how the *atg16l1^E226^* mice, which appear autophagy-defective, survive neonatal lethality. Neonatal lethality in *atg*5^−/-^ mice can be reversed by brain-specific re-expression of ATG5 [24]. These rescued mice (*atg5-null*) lack ATG5 and autophagy in non-neuronal tissues and develop multiple organ abnormalities with a phenotype closely resembling the *atg16l1^E226^* mouse described here. Both the *atg5-null* and *atg16l1^E226^* mice, survived neonatal lethality, but grew slowly compared to control mice, and have pronounced hepatomegaly and sarcopenia. This makes it possible that *atg16l1^E226^* mice described in our study survive neonatal lethality because they carry out a low level of autophagy in the brain. Interestingly, the LC3-I levels in the brain of *atg16l1^E226^* mice were the same as controls (Figures 9B and 9C). This was in contrast to peripheral tissues such as liver (Figure 5D), muscle (Figure 7B and C) and kidney (Figure 8B and C) where LC3 was elevated 3-4 fold. The preservation of control levels of LC3-I in *atg16l1^E226^* mice suggest that LC3 may be degraded in the brain by autophagy. This observation, and the striking similarity in phenotype with the neuronal-specific rescue of *atg5-null* mice described by Yoshii et al [24], suggests that the brain of *atg16l1^E226^* mice may compensate for the loss of autophagy arising from loss of the E230 residue required for WIPI2 binding. The size exclusion analysis of liver extracts in Figure 2 showed the elution of WIPI2 in high molecular-weight fractions in mice expressing full-length ATG16L1 or the CCD of ATG16L1 that retained glutamate E230 required for WIPI2 binding, but not in mice expressing the ATG16L1^E226^ CCD lacking E230. This provides an indirect assay for binding of WIPI2 to ATG16L1 when gel filtration was repeated for brain lysates (Figure 10). As seen in liver, ATG16L1 in control mice eluted in high molecular-mass fractions suggesting formation of a 300- to 600-kDa complex. These same fractions contained the ATG12–ATG5 conjugate, but surprisingly these fractions did not contain WIPI2, which eluted between 50 and 100 kDa (Figure 10). Similarly, the ATG16L1^E230^ and ATG16L1^E226^ CCDs formed high molecular-weight complexes and co-eluted with ATG12–ATG5 but were unable to move WIPI2 to high molecular-weight fractions. The profiles resembled that seen for the ATG16L1^E226^ CCD in liver that lacks the E230 glutamate required for WIPI2 binding (Figure 2). Taken together the results suggested that the binding of ATG16L1 to WIPI2 differs in brain compared to liver. This may provide the basis for a low level of autophagy in brain that allows the a*tg16l1^E226^* mice to survive neonatal lethality.

## Discussion

We have generated mice lacking the WD and linker domains of ATG16L1 to study the roles played by autophagy and LAP in maintaining tissue homeostasis *in vivo*. The *atg16l1^E230^* mice were unable to activate LAP, but retained glutamate E226 and E230 in the CCD required for WIPI2 binding and could therefore activate autophagy. These mice grew at the same rate as littermate controls, were fertile and did not have obvious defects in liver, kidney, brain or muscle homeostasis. This suggests that autophagy rather than LAP plays a major role in reducing tissue damage *in vivo*. The WD domain of ATG16L1 in higher eukaryotes including insects, nematodes, plants and humans contains over half the amino acids of the 66-kDa protein [21]. Gel filtration analysis suggested that full-length ATG16L1 formed a 300- to 600-kDa complex in liver and brain. The CCDs of the *atg16l1^E230^* and *atg16l1^E226^* mice eluted over a broad size ranging from 50–400 kDa suggesting multimeric assembly in the absence of the WD domain. These results are consistent with previous gel filtration analysis [21] demonstrating 600- to 800-kDa complexes of ATG16L1 in lysates of liver, brain and fibroblast cell lines, and the formation of additional smaller complexes ranging between 100 and 300 kDa in fibroblasts expressing ATG16L1 lacking the WD domain. Initial calculations based on gel filtration suggest formation of complexes containing 8 copies of ATG16L1 bound to the ATG12–ATG5 conjugate [21]. Later work analyzing the ATG16L1 complex by sucrose gradient sedimentation suggests ATG16L1 exists as a dimer [27]. This discrepancy in size estimation could be explained if ATG16L1 adopted an elongated conformation in solution accelerating its elution from gel filtration columns. Regardless of the precise size of complexes formed by ATG16L1 and the CCDs of the *atg16l1^E230^ and atg16l1^E226^ mice*, all three co-eluted with ATG12–ATG5. This increase in apparent size of the 72 kDa ATG12–ATG5 conjugate suggests the conjugate binds all 3 CCDs via the N-terminal ATG5 binding domain. The binding of WIPI2 to ATG16L1 requires glutamate E230 in the CCD, which is absent in the CCD of the *atg16l1^E226^ mice*. The lack of elution of WIPI2 in high molecular-weight liver fractions containing the CCD of the *atg16l1^E226^* mouse suggested that loss of glutamate E230 results in reduced WIPI2 binding *in vivo*.

The WD domains of ATG16L1 represent as much as a third of the total protein. It is perhaps remarkable that this complex of 7 bladed β-propellers, which are thought to provide a platform for protein-protein interactions important for autophagy, play little role in maintaining tissue homeostasis ‘in vivo’. Instead, evolution appears to have confined this important role to the N-terminal ATG5 binding and CC domains conserved through to the yeast ortholog Atg16 [28]. Binding of ATG16L1 to WIPI2 requires glutamate residues E226 and E230 in the ATG16L1 CCD, which bind arginine R108 and R125 exposed on the surface of WIPI2. Deletion of either E226 or E230 in ATG16L1 abrogates binding to WIPI2, and expression of WIPI2 lacking R108 and R125 is unable to reconstitute LC3 recruitment to phagophores in WIPI2-depleted cells [18]. Our study shows that the integrity of this WIP2 binding site within the short N-terminal domain is critical for autophagy *in vivo*, because loss of one amino acid, E230, required for WIPI2 binding, results in loss of autophagy and results in multiple tissue abnormalities.

Macrophages and fibroblasts cultured from mice lacking the WD domain were unable to activate LAP. This is in agreement with previous work [22], showing that the WD domain is required for several noncanonical pathways that recruit LC3-II to endocytic compartments. These include newly formed macropinosomes, phagosomes containing latex beads or apoptotic corpses, endosomes swollen by monensin or a combination of ammonium chloride and the vacuolating toxin A from *Helicobacter pylori*, and endosomes targeted by the M2 proton channel encoded by influenza virus. Furthermore, loss of the WD domain from ATG16L1 in dendritic cells results in reduced secretion of TNF and IL1B in response to CLEC4N/Dectin2 signalling [23] and reduced antigen presentation by dendritic cells [22]. The requirement of the WD domain for LAP suggests that the scaffold provided by the WD domains of ATG16L1 has evolved a specialized role, independent of autophagy, to ensure the quality control of endocytic pathways by conjugating LC3 to phagosomes containing pathogens or apoptotic cells, or endocytic compartments showing signs of damage. This is supported by the observation that the WD domain of ATG16L1 binds NOD-like receptors [29], MEFV/TRIM20 [30], TMEM59 [31] and EVA1A/TMEM166 [32], which are important in pathogen recognition. Our observation that mice remain viable and maintain tissue homeostasis over long periods in the absence of the WD domain suggests that LAP does not play an essential role in preventing tissue damage *in vivo*. LAP in phagocytic cells requires RUBCN and CYBB/NOX2 [3] and mice with *Lyz2/LysM-cre*-targeted disruption of either of these genes in macrophages, monocytes and neutrophils, grow slowly. We did not observe slowed growth for the *atg16l1^E230^* LAP-defective mice suggesting that loss of LAP from all tissues allows mice to compensate for loss of LAP in phagocytic cells. Alternatively, the deletion of the WD domain, and loss of RUBCN and CYBB/NOX2, inactivate LAP by different mechanisms. ATG16L1 is downstream of RUBCN and CYBB/NOX2, and it will be interesting to determine if the WD domain interacts with RUBCN or UVRAG or components of the CYBB/NOX2 complex. Defective clearance of apoptotic cells following LAP deficiency resulting from lack of CYBB/NOX2 or RUBCN in phagocytic cells predisposes mice to an autoimmune disease resembling systemic lupus erythematosus [4]. At 52 wk, *rubcn^−/-^* mice have increased levels of antibody against double-stranded DNA, anti-nuclear antibodies and deposition of immune complexes in the kidney. Our analysis of LAP-deficient *atg16l1^E230^* mice at 20–24 wk did not show elevated cytokines or anti-nuclear antibodies (not shown), but this does not preclude development of lupus-like autoimmunity as they age further.

MEFs cultured from *atg16l1^E226^* mice were unable to activate autophagy in response to starvation, and the mice showed accumulation of SQSTM1 *in vivo*. These mice grew slowly, were infertile and showed defects in liver, brain and muscle homeostasis, and neurological defects similar to those reported for mice with analogous tissue-specific loss of autophagy genes. These mice again highlight the importance of autophagy for maintaining tissue health *in vivo*, but, surprisingly, the *atg16l1^E226^* mice were able to survive neonatal lethality. These mice therefore differ from mice with systemic deletion of *Atg3, Atg5, Atg12 or Atg16l1*, which die within hours of birth because they cannot compensate for loss of placental nutrition [12,14,15]. This suggests that the truncated CCD^E226^ may provide functions that are missing when *Atg16l1* is deleted completely. The CCD^E226^ retains the ATG5 binding domain allowing the CCD ^E226^ to bind the ATG12–ATG5 conjugate and assemble into an ATG12–ATG5-ATG16L1 CCD^E226^ complex. This complex may maintain E3 ligase activity and be able to facilitate lipidation of LC3 and autophagosome formation at a low level. Evidence for a low level of autophagy is provided in Figure 3A where accumulation of SQSTM1 in MEFs from *atg16l1^E226^* appeared lower than following complete loss of ATG16L1, and there was a feint band for LC3-II. In addition, the requirement for WIPI2 binding to ATG16L1 to initiate autophagy may differ between brain and peripheral tissues. Support for this is provided by gel filtration analysis which suggested that binding of WIPI2 to the E230 glutamate residue in the CCD of ATG16L1 occurred in liver, but binding was much weaker in brain.

The phenotype of the *atg16l1^E226^* mouse was very similar to the *atg*5-null mouse described by Yoshii et al [24] where ATG5 expression was restored in the brain of *atg5^−/-^* mice. In common with *atg16l1^E226^* mice, the *atg*5-null mouse survived neonatal lethality but grew slowly and showed SQSTM1 accumulation in peripheral tissues, particularly liver and muscle. The *atg5*-null mice were sterile and have the pronounced hepatomegaly seen in *atg16l1^E226^* mice. This makes it possible that a low level of autophagy in the brain of *atg16l1^E226^* mice allows them to survive neonatal lethality. Indirect evidence for this is provided by the observation that LC3-I levels were not raised in the brains of *atg16l1^E226^* mice (Figure 8B) compared to muscle (Figure 6B), liver (Figure 4D) and kidney (7B). The truncated CCD^E226^ is able to protect against neonatal lethality, but not able to provide long-term protection against neurological defects. This phenotype is similar to the mice lacking *ATG*7 in the central nervous system [26] where neurons accumulate SQSTM1 inclusions and ubiquitin, and Purkinjie cells and pyramidal neurons are lost from the cerebellar cortex.

## Materials and methods

### Construction of the targeting vector

A P1 artificial chromosome (PAC) mouse genomic DNA library (Source BioScience, 711_RPCI21mPAC) was screened with a 0.5-kb Xho I-Sal I fragment obtained from the *Atg16l1* IMAGE cDNA clone 6,813,377/AV130 A2 (Source BioScience). A 10-kb XhoI and a 13-kb BamHI fragment, containing exons 1–10 of *Atg16l1*, were subcloned into pBSIISK- (Agilent, 212,206). Two stop codons and restriction sites for NheI, XhoI, EcoRV and EcoRI were introduced into exon 6 after amino acid position 230 by PCR. Two 1.5-kb fragments were generated using primer pairs CCAAATCCAGGTACCTCTCAG.seq in combination with ATCCTCGAGATCGATGCTAGCCTACTATTCCTTTGCTGCTTCTGCAAG.rev and ATCGATCTCGAGGATATCGAATTCCCTCTACCTGTTGAACAGTG.seq in combination with CCTGGCCCGGGCATGATAATG.rev, followed by an annealing PCR of the 2 fragments using primers CCAAATCCAGGTACCTCTCAG.seq and CCTGGCCCGGGCATGATAATG.rev to generate a 3-kb fragment, which was cloned into a SmaI-cut pUC19 vector (Addgene, 50,005). The bovine growth hormone polyadenylation site (bGH-pA; derived from pPGK-Cre-bpA, Addgene, 11,543) was cloned into the blunt-ended XhoI site, followed by cloning the blunt-ended PGK-Neo-frt cassette [33] into EcoRV. A KpnI-SmaI fragment of an 8.7-kb SacI-SmaI fragment, generated from the 10-kb XhoI and 13-kb BamHI genomic clones, was then exchanged with the modified exon 6-containing genomic PCR fragment.

### Embryonic stem cells and generation of homozygous mice

Embryonic stem cells were cultured as described previously [34]. R1 cells (2x10^7^) [35] were electroporated with 30 µg NotI-linearized targeting vector as described. G418-resistant clones were screened by Southern hybridization for homologous recombination. Positive clones were expanded, re-analyzed by Southern blot analysis and PCR, and injected into C57L/B6 blastocysts. Highly chimeric male founder mice were obtained, which were crossed with C57LB/6 females to obtain heterozygous F1 offspring. The neomycin cassette was removed by crossing F1 offspring with FlpO transgenic mice [36] and mice were subsequently crossed onto a C57L/B6 background. Genotype analysis was performed using primers CAAATATGCCTTCAGAACTG and GCTGTAGTTCCAATCCCTAA, resulting in 290-bp and 640-bp fragments for wild-type and mutant mice, respectively.

### Mice

These studies used adult male and female mice of approximately 2–3 months of age from the first cross of 129 and C57BL/6. All experiments were performed in accordance with UK Home Office guidelines and under the UK Animals (Scientific procedures) Act1986. The growth rate of these mice was estimated by recording weights each week. Mice were killed by schedule-1 procedures and dissected to harvest brain, liver, gastrocnemius muscle and kidney. The weights of the organs were recorded before fixing or freezing.

### Cells and cell culture

MEFs were generated by serial passage of cells taken from mice at embryonic day 13.5 and cultured in DMEM (ThermoFisher scientific, 11,570,586) with 10% FCS. BMDMs were generated from bone marrow isolated from femur and tibia flushed with RPMI 1640 (Sigma, R8758). Macrophages were generated by culturing adherent cells in RPMI 1640 containing 10% FCS and CSF1/M-CSF (Peprotech, 315–02; 30 ng/ml) for 6 d. Macrophage populations were quantified by FACS using antibodies against FCGR3/CD16-FCGR2B/CD32, ADGRE1/F4/80 and ITGAM/CD11b (BioLegend, 101,320, 123,107).

### Autophagy and LC3-associated phagocytosis

Autophagy was activated by incubating cells in HBSS (ThermoFisher, 11,550,456) to create starvation for 2 h at 37°C. LAP was stimulated in bone marrow-derived macrophages by Pam3CSK4-coupled beads. Carboxyl-modified beads (polybead carboxylate 3.0 µm, 09850) were conjugated with Pam3csk4 (invivogen, TLRL-PMS) by following the manufacturer’s protocol (Bangs Laboratories, Inc. Tech Note 205, III.). Pam3CSK4-coupled beads were added to macrophage cultures at a ratio of 10:1 (bead/cell) for 1.5 h before being fixed and the location of LC3 analyzed by immunofluorescence microscopy.

### Tissue western blotting

Dissected tissue was snap-frozen in liquid nitrogen, ground to a fine powder under liquid nitrogen and lysed in RIPA buffer (150 mM sodium chloride, 1% TritonX-100 [Sigma, P1379-1L], 0.5% sodium deoxycholate [Sigma, D-5670], 0.1% sodium dodecyl sulfate [Fisher Bioreagents, BP166-500], 50 mM Tris, pH 8.0) containing protease (Sigma, P8340) and phosphatase (Sigma, P5726) inhibitors followed by homogenization and freeze thaw. Samples were clarified by centrifugation (10,600 g, 10 min at 4°C). Supernatants containing ~10 ug protein were boiled in Laemmli buffer followed by SDS-PAGE using 4–12% gradient gels (Expedeon, NBT41212). The resolved proteins were electro-blotted onto nitrocellulose membrane (Bio-Rad, 1,620,115), blocked (5% skimmed milk in 1X TBS [50mM Tris (pH 7.5), 150mM NaCl], 1 h, room temperature) and then probed first with appropriate primary (ATG16L1 [MBL, M150-3], SQSTM1/p62 [Abcam, ab91526], GAPDH [Abcam, ab9482] and LC3A/B [Cell Signalling Technology, 4108]) and then secondary antibody (Cell Signalling Technology, 7074S and 7076S). Blots were visualized by exposure to Supersignal West Pico chemiluminescent substrate (ThermoFisher Scientific, 34,080). Bands were quantified through ImageJ (NIH, USA), analyzed (unpaired t-test) and plotted via GraphPad prism 7 software.

### Cell western blotting

Protein was extracted using M-PER (ThermoFisher Scientific, 78,501) with complete protease inhibitor cocktail (Sigma, 04693159001) for 30 min on ice. Samples were clarified by centrifugation (10,600Xg, 10 min). From the supernatants, protein concentrations were determined using the BCA protein assay system (ThermoFisher Scientific, 23,225) according to the manufacturer’s protocol. Protein (20 µg) was separated on a precast 4–12% gradient SDS-PAGE gel (Expedeon, NBT41212) and transferred to immobilon PVDF (Millipore, IPFL00010) for blotting. Membranes were probed using antibodies for ATG16L1 (MBL), SQSTM1/p62, (Abcam), LC3A/B and ACTB/actin (Sigma, A5441). Primary antibodies were detected using IRDye labelled secondary antibodies (LI-COR biosciences, 926–32,211, 926–68,020) at 1∶10,000 dilution. Proteins were visualized using the Odyssey infrared system (LI-COR).

### Gel filtration chromatography

Freshly dissected livers and brains were suspended in cold phosphate-buffered saline (Oxoid, BR0014G) containing protease inhibitors (Roche, 05892791001) and homogenized using a Dounce homogenizer. Particulate material was removed by sequential centrifugation at 100Xg and 13,000Xg (4°C) for 20 min. The supernatants were clarified by ultracentrifugation (100,000Xg, 4°C, 1 h) and analyzed by the gel filtration using an ENrich^TM^SEC 650 (Bio-Rad, 780–1650,) column and AKTA purifier (GE Healthcare) to collect 0.5-ml fractions. Fractions were analyzed by SDS-PAGE followed by western blotting on PVDF membranes probed for ATG16L1, ATG5 (Abcam, ab108327) and WIPI2 (Abcam, ab101985). The void volume (Vo) was estimated using Blue dextran (Sigma, D4772-1VL) and the elution volumes (Ve) of molecular size standards (Sigma, MWGF1000) allowed the determination of V_e_: V_0_ ratios to create a standard curve.

### Cytokine assays

Serum from young (2–3 months) and aged (5–6 months) mice were analyzed for cytokines using ProcartaPlex^TM^ Simplex Immunoassay kits (ThemoFisher Scientific, EPX01A-26,015–901, EPX01A-20,603–901, EPX01A-26,004–901, EPX01A-26,005–901, EPX01A-20,607–901 and EPX01A-26,002–901) by following the manufacturer’s instructions. Samples were read on a Luminex ^TM^ 100/200^TM^ instrument (Luminex Corp.).

### Histochemistry

Dissected tissues were fixed in 10% neutral buffer formalin (Sigma, HT501128), dehydrated, paraffin embedded and sectioned (5-µm thickness) prior to staining in hematoxylin and eosin (H&E). For immunohistochemistry deparaffinized and rehydrated sections were subjected to microwave-based antigen retrieval in citrate buffer (~ 0.24% trisodium citrate dihydrate, ~ 0.038% citric acid, in water). The sections were then incubated in hydrogen peroxide buffer (10% H_2_O_2_ in methanol) to mask any background peroxidase activity followed by treatment with blocking solution (10% goat serum [Gibco, 16,210–072], 0.30% Triton X-100 in PBS). Sections were stained with appropriate primary (anti SQSTM1/p62 antibody, anti MKI67/Ki67 antibody [Abcam, ab66155], anti ITGAM/Cd11b antibody [Abcam, ab133357]) and secondary antibodies (anti rabbit-HRP; Dako, K4003). The signals were developed with chromogen buffer (Dako, K3468). Tissue sections were mounted using cover glass and mounting medium (Neomount; Merck, 1,090,160,100) and imaged using 20X and 40X objectives on a bright-field microscope (Zeiss). The images were analyzed using Axio Vision software (cell circumference measurement, Axio Vision SE64 Rel. 4.8) and ImageJ software (cell counter plugin, NIH, USA). The obtained data were analyzed (unpaired t test) and plotted through GraphPad prism 7 software.

### Fluorescence imaging

Cells grown on glass coverslips were fixed at −20°C in ice cold methanol for 7 min, then blocked in 5% goat serum, 0.3% Triton X-100 in PBS (Sigma, G9023; X100). Cells were incubated with anti LC3A/B (Cell Signalling Technology, 4108; 1:500 in 1% BSA [Europa Bioproducts Ltd., EQBAH62-1000], 0.3% Triton X-100 in PBS). Washed cells were incubated with secondary antibody anti-rabbit-Alexa Fluor 488 (Life Technologies, A11008) and counterstained with 4ʹ, 6 diamidino-2-phenylindole (DAPI; ThermoFisher Scientific, 10,116,287) and mounted on slides with Fluoromount-G from Southern biotech (ThermoFisher Scientific, 15,586,276). Cells were imaged on a Zeiss Imager M2 Apotome microscope with a 63X, 1.4 NA oil-immersion objective using 365 ± 40 nm excitation and 445 ± 25 nm emission for DAPI, 470 ± 20 nm excitation and 525 ± 25 nm emission for LC3. Images were obtained using a Zeiss Axioplan microscope with bright field.

### Statistics

Unpaired t test was employed for the data analysis across all the experiments. The data were analyzed and plotted through GraphPad prism 7 software.

### Microscopy

All the fluorescent imaging was carried out using an Apotome microscope from Zeiss, fitted with 63X objective. All the immunohistochemistry for bright-field imaging was carried out using an Axioplan 2 microscope from Zeiss, fitted with 20X, 40X objectives and a colored Axio Cam HRc camera from Zeiss.
