# Supplementary material for: The ATG5-binding and coiled coil domains of ATG16L1 maintain autophagy and tissue homeostasis in mice independently of the WD domain required for LC3-associated phagocytosis
Source: Autophagy. 2018 Nov 7;15(4):599–612. doi: 10.1080/15548627.2018.1534507 (PMC6526875; doi:10.1080/15548627.2018.1534507)
Supplement: Supplemental Material [file kaup-15-04-1534507-s001.docx]

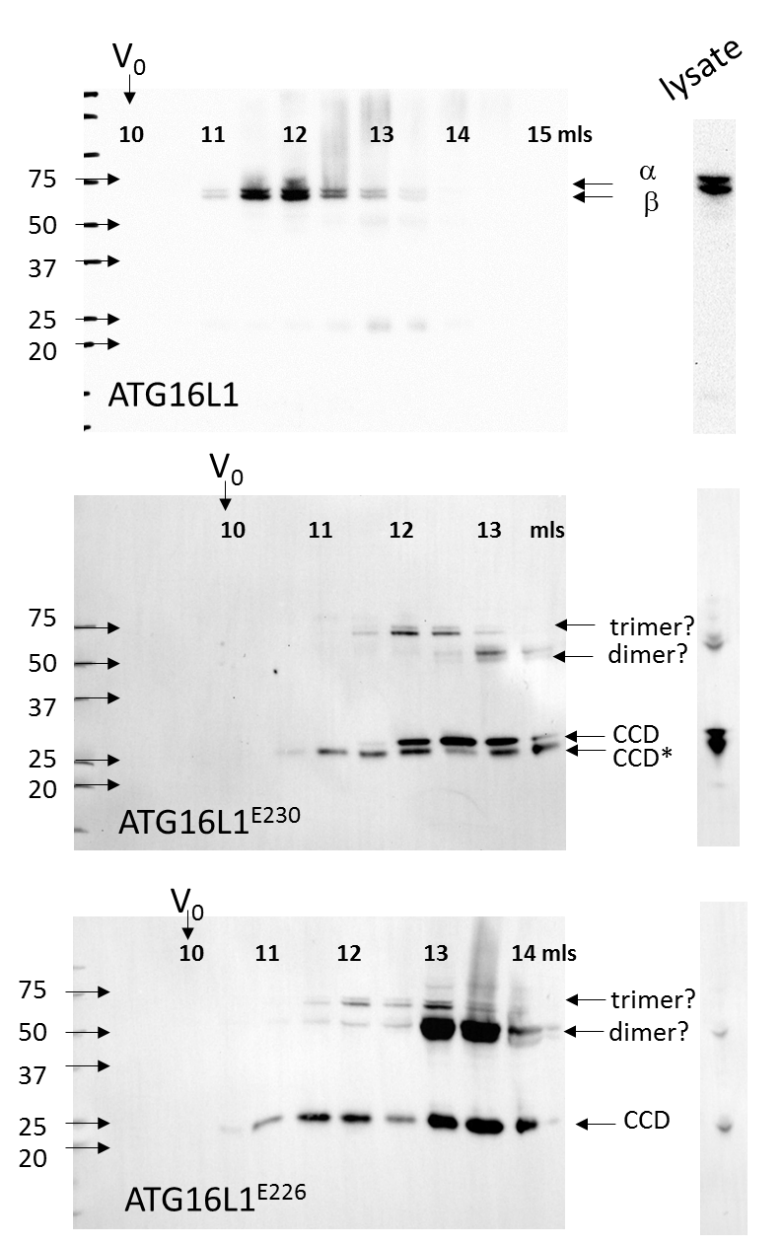


**Figure S1.** Analysis of ATG16L1 in liver lysates by gel filtration. Liver homogenates were clarified by ultracentrifugation and analyzed by gel filtration (see Materials and Methods). The images on the left show immunoblots of 0.5-ml fractions probed for ATG16L1 (MBL, M150-3) compared to whole tissue lysates (right). Migration of molecular mass standards during SDS-PAGE are shown (kDa). Immunoblotting of gel filtration fractions or whole lysates from control livers indicated major reactive proteins migrating at 65-70 kDa, the expected sizes of the α and β forms of ATG16L1. Analysis of *atg16l1^E230^* and *atg16l1^E226^* livers showed a doublet at 25 and 27 kDa, and bands at 50 kDa and 75 kDa. These are not seen in control liver suggesting they do not represent nonspecific binding of the antibody to liver proteins. The putative trimers elute faster (11.5-12.5 ml) than the dimers (13.0-14.0 ml). Their size is consistent with the formation of dimers and trimers of the CCD formed during the preparation of liver lysates that are not completely dissociated during SDS PAGE. The 25-kDa (CCD*) band may represent a proteolytic product of the 27-kDa CCD.


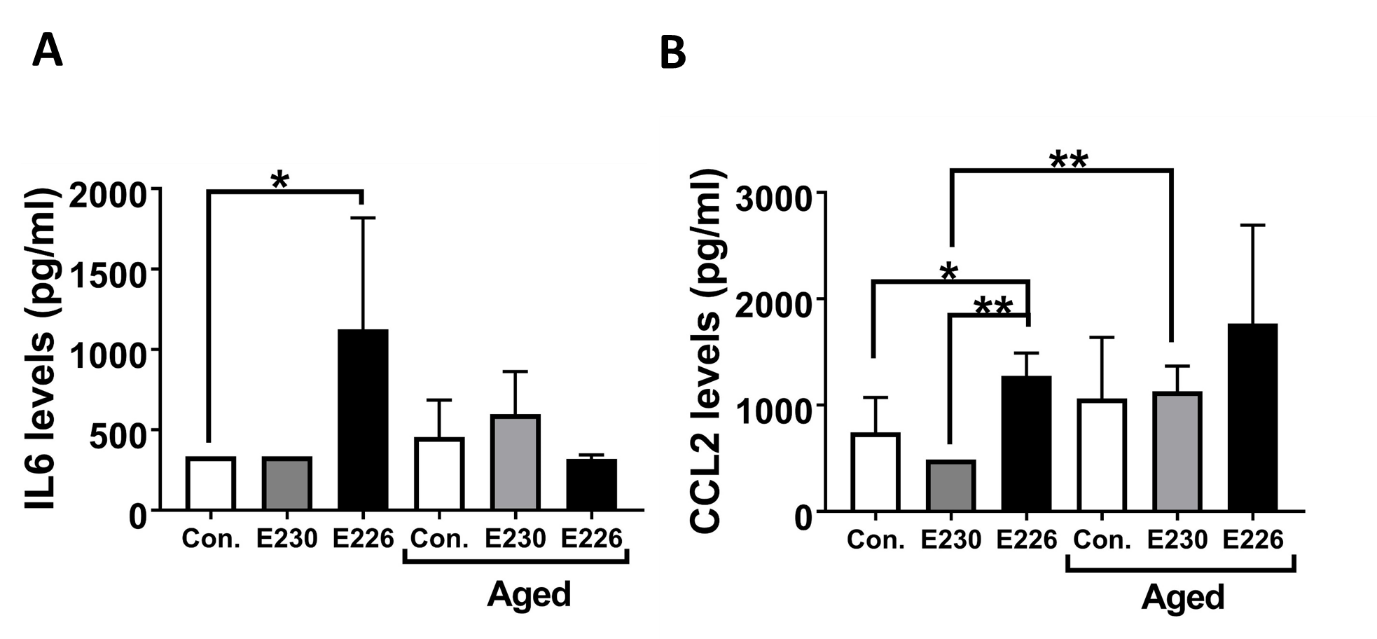


**Figure S2.** Analysis of cytokine levels in serum. Serum from young (8-12 weeks) and aged (20-24 weeks) control, *atg16l1^E230^* and *atg16l1^E226^* mice were analyzed for IL1B, IL12 (p70), IL13, TNF, IL6 and CCL2/MCP1 using ProcartaPlex^TM^ Simplex Immunoassay kits. IL6 and CCL2 levels were found to be different across the indicated groups. n=5 (control young), n=6 (control aged), n=3 (E230 young and aged), n= 2(E226 young), n=3 (E226 aged). Data across littermate control mice for E230 and E226 were pooled. Statistical analysis was done by unpaired t test. Error bars represents ± SEM. **-P<0.01, *-P<0.1. Serum levels of IL1B, IL12 (p70), IL13, and TNF in *atg16l1^E230^*, *atg16l1^E226^* and their littermate controls were below the detection limits of the assay at 8-12 and 20-24 weeks. The lower limits are shown in parentheses after the indicated cytokine IL1B (<75 pg/ml), IL12 (p70) (<243.75 pg/ml), IL13 (<156.25 pg/ml), and TNF (<200.0 pg/ml).


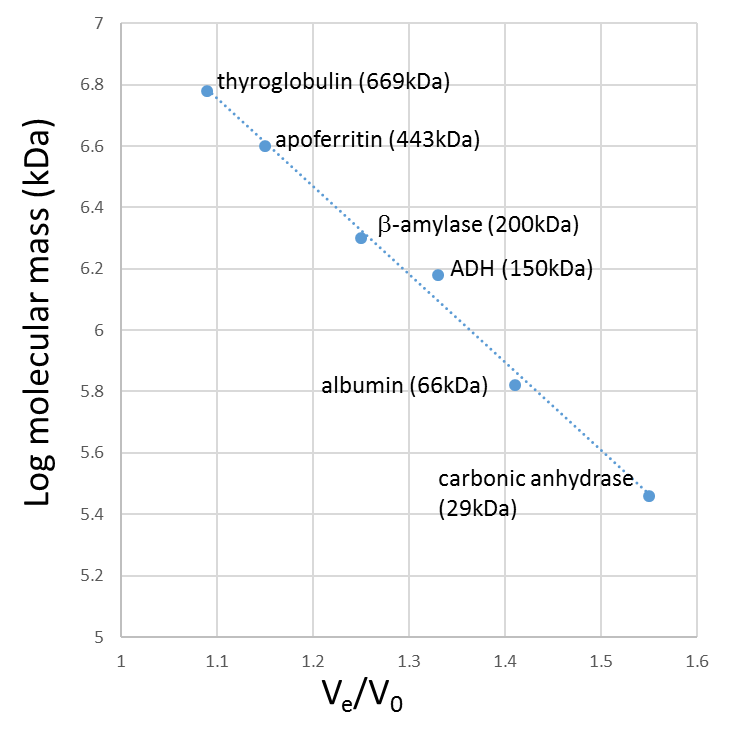


**Figure S3.** Calibration of gel filtration column. Protein standards were eluted from an ENrich^TM^SEC 650 column (Bio-Rad) using an AKTA purifier (GE Healthcare) to collect 0.5-ml fractions. The void volume (V_o_) was estimated using Blue dextran and the elution volumes (Ve) of the indicated molecular size standards (Sigma, MWGF1000) allowed V_e_:V_0_ ratios to create the standard curve.
